# American Association for Accreditation of Ambulatory Surgical Facilities (AAAASF) History: Its Role in Plastic Surgery Safety

**DOI:** 10.1093/asjof/ojz008

**Published:** 2019-04-02

**Authors:** Robert Singer, Geoffrey R Keyes, Foad Nahai

**Affiliations:** 1 The University of California, San Diego (UCSD), San Diego CA; 2 Emory University School of Medicine, Atlanta, GA; 3 Division of Plastic Surgery, Keck School of Medicine, University of Southern California, Los Angeles, CA

## Abstract

From its origin cosmetic surgery was performed in facilities which were neither certified nor regulated. Recognizing that there was no formal oversight of facilities, a group of plastic surgeons saw the need to develop an accreditation program. This eventually evolved into the American Association for Accreditation of Ambulatory Plastic Surgery Facilities. The organization was started to implement and maintain a voluntary inspection accreditation program for qualifying surgical facilities. Its focus was to educate plastic surgeons on safety and became recognized as the gold standard for accreditation. Seeing the need for similar standards for all surgeons, it morphed into the American Association for Accreditation of Ambulatory Surgery Facilities (AAAASF). Comprehending that accreditation was in everyone’s best interest, AAAASF developed educational formats for plastic surgeons, testified at the US Congress suggesting potential ways that oversight of facilities could improve patient safety, functioned as a resource to numerous states in developing guidelines for oversight of facilities, continued to update its standards, and extended its accreditation program internationally. Recognizing the value of accreditation, proven by AAAAASF’s extensive database from its Internet-Based Quality Assurance Program, the American Society for Aesthetic Plastic Surgery (ASAPS) and the American Society of Plastic Surgeons (ASPS) mandated that its members operate only in accredited or licensed facilities. Numerous studies documenting the safety of accredited plastic surgical facilities from AAAASF’s extensive quality assurance and peer-review reporting program are cited. AAAASF played a significant role and will continue to do that in producing better, safer environments for outpatient surgical procedures.

Date back to the early and mid-1900s, plastic surgery, primarily cosmetic procedures, were undertaken in hotel rooms and subsequently in office facilities with no oversight or regulations.^1^ Sadly in parts of the world and even here at home, cosmetic procedures continue to take place in less than ideal settings.^2^ The Joint Commission on Accreditation of Hospitals (JCAH) was created in 1951 and the Accreditation for Ambulatory Health Care (AAAHC) was started in the 1970s to accredit regional ambulatory centers. Those organizations did not provide accreditation of freestanding office-based surgical facilities. While there developed a tremendous increase in the numbers of surgical procedures, plastic and others, performed on an ambulatory basis, the federal regulatory bodies lagged behind the changes in the provisions of health care: In 1980, there were over 275 recognized ambulatory surgical centers, in 1990 nearly 1450 centers, there were 3700 in the United States in 2003, and an estimated over 5500 in 2014. It was estimated that about 65% of all surgical procedures were undertaken on an outpatient basis in 2014, while in 1980, it was estimated to be only 15%.^3^

This trend and the need for improving patient safety was recognized by organized plastic surgery. In 1977, the American Society of Plastic and Reconstructive Surgeons (ASPRS), which subsequently renamed itself the American Society of Plastic (ASPS), formed a committee that included Mr. Edward Stygar, Jr. to establish and operate an office-based surgery accreditation program. This committee conceived and established the American Association for Accreditation of Ambulatory Plastic Facilities (AAAAPSF) to guarantee the quality of outpatient plastic surgical facilities. In 1980, Mr. Stygar, who had experience in managing other accreditation programs, was appointed the Executive Director. A board was created by Dr. Edward Truppman to help facilitate the development of an accreditation and inspection program for plastic surgery office-based facilities. The board worked hard and was able to create both a standards and inspection manual as well as a safety checklist questionnaire. AAAAPSF initially established standards for single specialty ambulatory surgery centers (ASC) operated by surgeons who were board certified by the American Board of Plastic Surgery.^4^ In 1992, AAAASPF morphed into the American Association for Accreditation of Ambulatory Surgery Facilities (AAAASF), recognizing the need for similar standards for all American Board of Medical Specialties (ABMS) surgeons who operated in single surgical or multispecialty ASCs and were board certified, practicing within the scope of their specialty.^5^

Plastic surgeries are classified into three types: Class A, minor plastic surgery procedures performed under local, regional, or topical anesthesia; Class A/B, minor or major plastic surgeries performed under intravenous or parenteral sedation, analgesia, or dissociative drugs; and Class A/B/C, major plastic surgeries performed under general anesthesia requiring external support of vital body functions. At that time, no facilities were accredited for elective procedures in major body cavities.

Office-based facilities that participated and sought accreditation did so voluntarily. Inspections initially began in 1979, with the first accreditations awarded by the board of directors to 103 facilities in 1980. Initially 40% of the applying facilities were approved, 55% were granted provisional status, and 5% were denied accreditation. Interestingly, the President of the ASPRS as well as members of the AAAASF Board were granted only provisional approval on their first facility examination and had to correct minor deficiencies until they ultimately received accreditation.^5^

The ASPRS was started as a not-for-profit professional association devoted to the implementation and maintenance of a voluntary inspection accreditation program of qualifying ambulatory surgical facilities by a cadre of volunteer inspectors. The initial board of directors was appointed by the board of directors of ASPRS, who initially opposed any representation from other plastic surgery organizations. Subsequently, there was progressive representation of the various plastic surgical societies, both regional and nationally through the ASAPS. ASPRS funded the initial start-up of the organization and was eventually reimbursed by AAAAPSF.

The net income from November 1980 to December 31, 1981 was $8,817.81 based on a total income of $80,289.74. The initial cost of accreditation was $450 per year and only $300 for a Class A facility.

Part of the initial board of directors consisted of Drs. Truppman, Rex Peterson, Sherrell Aston, Dale Dubin, Arthur Ship, William Porterfield, Eugene Worton, George Hoffman, David Gilbert, and Frederick McCoy. Dallas Whaley, the Executive Director of ASPRS, was named the first Executive Secretary. Dr. Gus Colon who followed Dr. Truppman as president, John Krause, Charles Vinnick, John Williams, Fred Grazer, Simon Fredricks, Stan Klatsky, Richard Walden, Ray Elliot, Paul Schnur, and Robert Reeder were all added to the board at various times during the first decade of its existence.

Drs. Dan Morello, Robert Singer, Ron Iverson, Mike McGuire, Alan Gold, Jim Yates, Larry Reed, Harlan Pollock, Geoffrey Keyes, and Foad Nahai all were added during that initial decade or at various later times to the Board and each eventually became President.

## EDUCATION

As early as 1983, Dr. Truppman reported that the general cost for services in ambulatory plastic surgery facilities were generally 70–80% lower than costs for similar procedures performed in the hospital.^6^ At that time, 80% of private practicing plastic surgeons performed ambulatory surgery primarily in office settings.

To educate the plastic surgery community about patient safety in office-based surgical facilities, Ambulatory Surgery Symposia were scheduled in conjunction with the ASAPS meeting in 1981 and in several subsequent years. In 1985, AAAASF appealed to the various plastic surgery societies to provide educational programs on ambulatory surgery which they were not adequately doing at that time. Educational teaching courses, panels, webinars, and free-standing sessions on patient safety in ambulatory surgery have been presented by AAAASF since then.

The American Association for Accreditation of Ambulatory Surgery Facilities Education Foundation (AAAASF-EF) was established in 2001, with Dr. Singer as its President. That proved to be a step forward in the improvement of ambulatory surgery education and to ensure progress in safety. In 2007, a national conference entitled “Patient Safety in the Office Based Surgery Setting” which was organized and chaired by Dr. Singer and Dr. Tom Russell, Executive Director of the American College of Surgeons, was held in Chicago. This landmark conference was developed by AAAASF-EF and included the other nationally recognized accrediting organizations: AAAHC, the Joint Commission, the Health Care Facilities Accreditation Program of American Osteopathic Association (AOA), American College of Surgeons (ACS), ASPS, ASAPS, American College of Foot and Ankle Surgeons (ACFAS), and the Federation of State Medical Boards (FSMB).

At that meeting, the first ever patient safety summit was held with more than 90 participants from some 30 associations. The conference was an enormous success and elevated AAAASF nationally which led to its recognition by surgeons, legislators, state, and national health agencies as the “gold standard” for accreditation.

Dr. Keyes presented data on over 1,000,000 procedures, obtained from the Internet Based Quality Assurance Program (IBQAP), that he conceived and developed at the request of Dr. Singer, during his Presidency of AAAASF in 1999. Dr. Keyes advocated creation of a standardized, internet-based data entry process to record patient demographic and procedural information and report patient outcomes. “The data must be free-flowing and shared,” he said.^4^ The other accrediting organizations did not have this type of data and decided not to be involved in this endeavor.

“We have no way of measuring safety risk outside of accredited facilities,” said Dr. Singer. “With some 95 percent of outpatient facilities operating without accreditation, this conference provided a breakthrough opportunity for national accrediting agencies and professional societies to work together in a progressive effort that will impact positively on patient safety. More can be done in the states as a coalition than alone.” He concluded the conference by stating that “Accreditation is in everyone’s best interest.”^4^

According to AAAASF’s prior-President Dr. Alan Gold, the conference uncovered some startling information and magnified the need for better patient outcome data in outpatient surgery. “Data on more than one million surgeries performed in accredited outpatient facilities show comparable or lower rates of morbidity and mortality than anticipated in the traditional hospital setting. We believe this demonstrates that adherence to AAAASF standards has improved patient safety,” Gold said.^4^

## INPUT TO CONGRESS

In June 1989, Drs. Truppman, Colon, Singer, and Mr. Ed Stygar (the Executive Director) were invited and testified in the hearing before the US Congress Subcommittee on Regulation of Business Opportunities and Energy of the Committee on Small Business House of Representatives. This committee hearing addressed “Cosmetic Surgery Procedures: Standards, Quality, and Certification of Non-Hospital Operating Rooms.”^7^

Dr. Truppman testified that AAAAPSF proposed to the American College of Surgeons:

1. Creation of a council of accreditation associations, especially ambulatory surgical facilities;
2. Development of tiered generic standards for all facilities, especially ambulatory surgical facilities;
3. Formulation of specific standards for each specialty when necessary.

Dr. Colon testified about the “concern of the unbridled proliferation of non-accredited surgical facilities across the country and the concern that they pose significant danger to the public because of sub-standard conditions. Some of these facilities are owned and staffed by physicians with little regard for even minimum standards of patient safety.”^7^ He testified when the triangle of morality, ethics, and professionalism, representing the guiding principles in all professions, particularly medicine, is over turned by greed, the patient is always the one who suffers. Dr. Colon felt that the public should be educated:

1. On the risks of surgery,
2. Qualifications of the surgeons,
3. The importance of facility accreditation.

He also stated that the FTC must do its part to protect the consumer and force regulations. “Without swift and thorough action on the part of the appropriate agency, many, many more innocent people will be victimized by greedy, unscrupulous physicians falsely bearing the title and mantle of plastic surgeons and taking refuge in unaccredited and substandard office surgical facilities.”^7^ It is amazing to see how prophetic that statement proved to be.

Dr. Singer recommended:

1. That Congress develop national standards governing both the office-based surgical facilities and the surgeons who practice in them which should consist of core generic standards that apply to all types of ambulatory surgical facilities.
2. Enforce these standards on a mandatory basis which can be accomplished by existing nationally recognized accrediting agencies. This recommended approach would avoid the need for additional bureaucracy, manpower, and funds.
3. Mandate peer review for physicians practicing in office-based facilities.
4. Require of malpractice insurance for physicians operating in their offices.
5. Ensure truth in advertising by requiring full disclosure of the physician’s training and limiting claims of board certification by those physicians certified by boards recognized by the American Board of Medical Specialties. In addition, doctors claiming board certification should be required to state the full name of the board that certified them.
6. Implement these mandates through national legislation and/or model statutes for each state.

Unfortunately, after getting significant media coverage, the Committee chaired by Representative Ron Wyden took no significant, subsequent action to improve patient safety.

## STATE ACTIVITY

State legislative activities were more productive than interacting with Congress. California, where much of office-based surgery started, became the first state to mandate accreditation for all outpatient facilities that administer sedation or general anesthesia. AAAASF was instrumental in the development of the California legislation (AB595) which was passed in 1995,^4,5^ and was implemented in 1996. AAAASF was also a valuable resource for subsequent laws and regulations that were adopted by numerous other states with regards to ambulatory surgery and Certificates of Need for facilities including: Florida, Georgia, New Jersey, Pennsylvania, Texas, Maryland, and Alabama. AAAASF Board members have continued to provide input to other state medical board and legislative bodies with regard to guidelines for office-based surgery and regulations.^8^

Legislation in New York required office-based facility accreditation in 2007. Despite 26 other states requiring similar accreditation, there was no consistency regarding their regulations or effective ways to ensure compliance. AAAASF was in the vanguard in providing input to New York State when legislation was passed to make office-based surgery accreditation mandatory for any facility utilizing moderate anesthesia or above for any of their procedures. Data were presented to the New York Commission responsible for medical regulations by Dr. Keyes, at a meeting attended by the JCAOH, AAAHC, and AAAASF representatives. The state regulatory agencies were uncertain as to whether they should require accreditation or licensure for their outpatient surgery centers. After seeing the data collected by AAAASF on outpatient procedures and unanticipated sequelae, they were convinced that accreditation was necessary. The other accrediting associations did not collect data. One would ask why all the other states that currently require no oversight have not implemented similar requirements.^9^

In 2008, AAAASF restructured including new board members from more diverse specialties after recognizing that over 50% of the accredited offices were nonplastic surgery facilities. Permanent board seats were established for Association of Peri-Operative Registered Nurses (AORN), American Association of Nurse Anesthetists (AANA) and the American Society of Anesthesiologists (ASA). It was felt that as more diversified medical specialties seek accreditation; their input was necessary to best serve the medical community and best protect the safety of patients.

AAAASF accreditation guidelines have been accepted by many State Departments of Health in lieu of state licensure, as well as being approved to inspect facilities for certification by Medicare. Because of continued disasters in nonaccredited and uninspected facilities across the country, AAAASF has been involved in ongoing dialog and interaction in California and other states legislatures. The Medical Board of California has consulted with AAAASF to modify regulations that ensure patient safety, including suggested guidelines about appropriate training for those performing the procedures. Finally, AAAASF also worked with the New York State Society of Oral and Maxillofacial Surgery to create an accreditation program.

## STANDARDS MODIFICATIONS

The AAAASF standards cover multiple areas and all must be complied with to achieve and maintain accreditation (Tables 1 and 2).^10^

**Table 1.** Evolution of AAAASF

1983: A peer review system was put in place.
1985: The participation of physicians from specialties other than plastic surgery in AAAASF-accredited facilities was discussed. At that time, the existing policy limited accredited facility use to board-certified plastic surgeons and occasional use by other specialties was permitted under certain circumstances.
1986: The inspection cycle was changed from 2 to 3 years.
1987: The Board opted to expand participation of use of the facilities by other surgical specialties.
1989: Dr. Colon was elected president following Dr. Truppman who held the position since the start of the organization.
1992: The first newsletter was developed by Dr. Iverson and distributed to member offices. The same year, the concept of the AAAAPSF accreditation umbrella was created to cover all American Board of Medical Specialties’ (ABMS) surgical specialties and ambulatory surgery centers, and, to reflect this expansion, the name of the organization was officially changed to AAAASF in 1994.
1993: A landmark was reached of having inspected 500 facilities, with 431 being active. Ambulatory surgery continued to grow. In 1999, ambulatory and outpatient settings accounted for over 70% of the 66.5 million surgical procedures billed to Medicare annually. That number outweighed the hospital inpatient settings by five to one.
1993: A brochure was developed for patients to explain accreditation and ongoing symposia were presented about accreditation. As ambulatory surgery continued to grow, the Board felt there was a need for a facility survey regarding mortality and complications in AAAAPSF-accredited facilities with the information compiled through an independent agency. The survey of complications and fatalities was funded by ASAPS, ASPRS, and ASERF.
1993: Reaching out to our Canadian colleagues, who also were interested in accreditation, the Board opted to approve the recognition of Canadian Boards that same year.
1998: AAAASF became a sponsoring organization of the American Board of Plastic Surgery (ABPS). The mission statement of AAASF was created: “The mission of the association is to develop and implement standards of excellence in quality patient care through an accreditation system for ambulatory surgical facilities and to create the public interest by providing accurate and timely information regarding surgery and ambulatory surgical facilities.”

AAAAPSF, The American Association for Accreditation of Ambulatory Plastic Facilities; AAAASF, The American Association for Accreditation of Ambulatory Surgery Facilities; ABMS, American Board of Medical Specialties; ASAPS, The American Society for Aesthetic Plastic Surgery; ASPRS, American Society of Plastic and Reconstructive Surgeons; ABPS, American Board of Plastic Surgery.

**Table 2.** Standards

Basic mandate operating room policy, environment, and procedures
Post-anesthetic care unit (PACU)
General safety in the facility
IV fluids and medications
Medical records
Quality assessment/quality improvement
Personnel
Anesthesia

There have been significant modifications of the standards since the origins of the organization and the standards and guidelines have been continually refined and updated to improve them. Developments in 1996 included adding an OSHA manual guideline to the accreditation book as well as modification of the Bylaws. These improvements made it possible for an ABMS board-certified anesthesiologist to own and direct a facility where surgeons performed surgery. In addition, appropriately trained life members of ASPRS and ASAPS were made eligible to act as inspectors.

In 1998, after recognizing that patient safety problems were occurring across the country because there were no appropriate guidelines for overnight postoperative stays in office-based surgical facilities, an AAAASF task force, chaired by Dr. Jim Yates, developed guidelines for overnight 23-hour stays. Those guidelines were subsequently adopted by the Medical Board of California.

In 2000, AAAASF developed guidelines for the delivery of anesthesia services. The basis of those guidelines was incorporated from the American Society of Anesthesiologists (ASA) standards. Dr. Jeff Apfelbaum, who eventually held the position of President of ASA, was the first anesthesiologist added to the AAAASF Board.^10^

Dr. Gold stated that our “Standards may be considered the strongest of any organization that accredits ambulatory surgery facilities, and that many consider them to be the “gold standard.” We recognize, though, that they need to be part of a “living document,” and continually re-evaluate and revise those Standards in the light of medical advances and changing legislative guidelines.”^4^

Most of the standards would be common to any specialty, not just plastic surgery. A ‘surgical pause” or “surgical time out” was added to the standards in 2008.^11^

## PLASTIC SURGERY SAFETY

Office-based surgery is more economical for the patient, less stressful than a hospital admission with the excessive and increasing amount of bureaucratic red tape, more private, offers greater convenience and more personalized care with a continuity of exposure to the same personnel. Patient safety should, however, always be primary, before convenience or cost. There were numerous advances in outpatient plastic surgery safety, including an awareness and education of physicians that operating in an accredited facility is not the only factor in achieving safe outcomes.^12^ Accreditation standards and regulations cannot ensure patient safety without a physician’s common sense and prudent judgement. There are a multitude of other factors: appropriate surgical training, excellent patient care preoperatively, intraoperatively and postoperative and a key aspect, appropriate patient selection.^13-19^ Not every patient is a candidate for office-based surgery!

A significant step toward improving safe outcomes was the introduction of intraoperative patient monitoring with the use of the pulse oximeter in the ambulatory aesthetic surgical facility, which was presented at the ASAPS Annual Meeting in 1987 and has been a standard of care since.^20^

In 1999, following Dr. Singer’s introduction of the concept of requiring accreditation for ambulatory surgical facilities at the Council of Plastic Surgery Organization (COPSO) and after lengthy discussions and education of the Board members of both ASAPS and ASPS by Drs. Iverson and Singer, both organizations recognized the need and value of accreditation. The two organizations mandated that members who utilized an office-based freestanding surgical facility must operate only in an accredited or licensed medical facility. All members had to comply by July 2002.^21^ Organized plastic surgery led the way in this arena and at that time it was the only surgical specialty that made this a requirement for membership in those key societies. Patient safety should be a priority of all specialties and all should embrace this concept. They could all significantly raise the bar of safety by mandating that all their members who utilize office-based freestanding surgical facilities must operate only in an accredited or licensed medical facility.

Because of concerns of safety, in 2003 AAAASF ruled that propofol (Diprovan) was classified as a general anesthetic and only administered in class C facilities under direct supervision of an anesthesiologist or CRNA.^22^ Numerous other concerns were addressed and incorporated into safety steps by AAAASF to educate of physicians through the years (Table 3).

**Table 3.** Steps to Improve Safety

• Guidelines for liposuction volume removal.^47^
• A protocol for dealing with malignant hyperthermia, and awareness of issues about hypothermia.^48^
• A required assessment for pregnancy pre-op was put in place and required discussion and documentation of pregnancy testing policy with each patient (2015).
• Information about avoidance and treatment of nausea and emesis.^49^
• A documented pre-surgical time-out check off list.^11^
• Adherence to the less than 24 hours stay guidelines for facilities keeping patients overnight.
• A mandatory reporting of untoward sequelae.
• Awareness and appropriate management of the patient with sleep apnea.
• An alert about latex allergies.
• Because of the international concern about Ebola and the fact that outpatient facilities treat many patients daily, the organization provided a facility preparedness checklist for Ebola, as well as facts about the virus in the United States.
• Beyond plastic surgery
o 2002: Privileges in AAAASF-accredited facilities were extended to board-certified anesthesiologists, or anesthesiologists that held hospital privileges for those same procedures being performed in an accredited facility, to provide pain management to their patients.
o With the growing number of office-based facilities offering a variety of nonsurgical services and ancillary procedures, AAAASF amended the standards to allow inclusion of highly trained, highly skilled physicians with ABMS certification in nonsurgical specialties who are performing procedures as delineated by their board certification, as staff members in an AAAASF-accredited office-based facility or ASC.
o At the same time, a change occurred in the composition of the Board of Directors to bring on representatives of multiple medical and surgical specialties as well as a public member.
• In 2003 AAAASF contracted with Online Labs, Inc. to completely recreate the website, revamp and improve the online Quality Assurance and Peer Review reporting program, and develop a web-based accreditation program. Under the guidance of Geoffrey Keyes, M.D., the Chair of the Technology and Quality Assurance Committees, the entire accreditation process was evaluated, improved, and simplified as AAAASF converted to the new system.
• As of May 2016, there was a total of 2417 accredited facility: 652 procedural, 1057 surgical, 33 International Dental and Surgical, 222 OPT, 236 RHC, 21 OMS, and 196 ASC. The international facilities exist in 11 countries. There was a huge growth in the RHC and RA/OPT accreditation programs. In 2016, Medicare review was completed and approved.

AAAASF, the American Association for Accreditation of Ambulatory Surgery Facilities; ABMS, American Board of Medical Specialties; ASC, ambulatory surgery centers.

One noteworthy addition was the incorporation of a standard that requires screening protocols for venous thromboembolism (VTE) risk factors. This standard was recommended by Dr. Keyes and originated from the peer-review and unanticipated sequelae reporting system in which every accredited facility participates.^23,24^ This was a pivotal moment for AAAASF, raising the standards for outpatient surgery highlighting its focus and commitment to patient safety.

Overall patient safety and assuring the well-being of patients and facility staff have been and remain the primary concern to AAAASF.

## IMPROVING INSPECTIONS

Until 1999 inspectors donated their time on a voluntary basis without reimbursement to hold down the cost of accreditation. In 1999, the organization started paying an honorarium upon request to inspectors of facilities because of the increasing number of facilities requiring inspection and the fact that this was no longer a single-specialty accrediting agency. In 2000, Dr. Gold, head of the Inspectors’ Committee, recommended and the Board approved that nurses could function as pre-inspectors and could be utilized as part of the inspection team.

A Medicare training program was developed for both Medicare inspectors and inspectees, and a Technology and Communications Committee was formed to streamline the efficiency of the central office and communication to the membership. To further improve the inspection process, the Education Committee, under the active chairmanship of Drs. Gary Brownstein and David Watts, continued to refine the three courses that the AAAASF offered: (1) The Initial Inspector’s Training Course; (2) The Medicare Inspector’s Training Course; and (3) Recertification for Current Inspectors.

While AAAASF developed a process to ensure patient safety in an outpatient setting, they recognized that the activity was time consuming and deserved an attempt at streamlining it for its members. A new website was created in August 2005 to help facilitate this followed several years later by a new surveyor’s website. This allowed ongoing certification of the surveyors and allowed for better dissemination of critical information regarding these surveys.

The AAAASF Quality Assurance Surveyors Oversight Committee was created and approved in 2009 to research, establish, and implement new quality oversight systems that would more carefully monitor and evaluate the quality of our inspections (surveys) process and our inspectors (surveyors methods of reviewing surveyors continue to be refined and it was required that surveyors be retrained every three years by either a webinar or on site to continue to maintain quality.

## PEER-REVIEW DATA

Valid data helped position AAAASF as the reliable resource about patient safety in ambulatory surgery. To address the question of patient safety in office surgery facilities, AAAASF commissioned a research project to:

1. Identify and quantify complications related to such operations,
2. To derive summary information about deaths that occurred during or immediately following such procedures,
3. To compare these mortality and morbidity data with outpatient data compiled by other entities/agencies

A landmark article published in 1997 covered data of 400,675 surgical procedures from January 1, 1989 to December 31, 1993 and showed the overall risk of significant complications was comparable in an office (plastic surgical facility) and in a free-standing or hospital ambulatory surgical facility.^25^

To obtain additional ongoing valuable data, the first iteration of the online peer-review system was developed in 1999 for AAAASF by Dr. Keyes at Dr. Singer’s request. Since 2001, all physicians operating in AAAASF accredited facilities were required to report all unanticipated sequelae every six months as part of the Quality Assessment/Quality Improvement Program. Dr. Dennis Thompson did a superb ongoing job as a Board member and chairing the critical Investigative Committee. This AAAASF peer review data system was the basis for several key articles about safety including:

- “Analysis of Outpatient Surgery Center Safety Using an Intranet–Based Quality Improvement and Peer Review Program”^26^
- “Mortality in Outpatient Surgery”^27^

Throughout the decade from 2000 to 2010, AAAASF continued to revolutionize data collection in ambulatory surgery. That ongoing effort alone set the organization far apart from other accrediting organizations and medical associations who did not have available data. AAAASF accumulated data on over 2.5 million procedures performed in their accredited facilities. For AAAASF accredited facilities, this solidified the recommendations and standards as being fact-based using solid statistics. Partly because of that verifiable data, in 2010 the US Centers for Medicare & Medicaid Services (CMS) announced the approval of deeming authority for AAAASF. The enormous amount of ongoing collected and analyzed data led to further publications.

“Outpatient Surgery and Sequelae: An Analysis of the AAAASF Internet-Based Quality Assurance and Peer Review Database,”^28^ a review published by Soltani et al in 2013 was the largest data set in ambulatory surgery (7,629,686 procedures on 5,416,071 patients, which included 5,525,225 plastic surgery procedures on 3,922,202 patients). Hematoma and infection were the most frequent complications.

The safety of accredited office-based facilities has been verified in other published articles: Outpatient plastic surgery has been shown to be safe, as shown by Byrd et al with a complication rate of less than 1% in 5316 consecutive cases.^29^ Additional publications have demonstrated complication rates between 0.33% and 0.7% with a mortality rate of 0.002%.^30,31^

CosmetAssure is an insurance program that covers the cost of unexpected major complications from 24 covered cosmetic surgical procedures in all 50 states in the United States. It has been prospectively collecting risk factor data for research purposes since 2008. They reported on 129,007 patients from 2008 to 2013 that patients operated on in office-based surgical suites had a lower rate of complications (1.3%) than in ambulatory surgical centers (1.9%) or in hospitals (2.4%).^32,33^ That does not mean that the risk factor is zero, since surgery of any kind including plastic surgery has potential risks that are not always totally predictable, and complications can occur in the best of facilities and in the hands of even the most superb surgeons. The conclusion in that report is similar to the reports based on AAAASF’s data that office-based plastic surgical facilities are safe and the risks are low when the facilities are accredited, the surgery is performed by a board certified or eligible plastic surgeon and there is proper patient selection.

Dr. Hector Vila, an anesthesiologist in Florida, was initially critical of the level of safety in office-based facilities and was the lead author of a 2003 *Archives of Surgery* article on office-based surgery suite (OBSS) safety that found office-based surgical suite (OBSS) surgery 10 times riskier than surgery performed in ASC; a study that opened the discussions on safety concerns of office-based surgical procedures in Florida.^34^

Dr. Vila joined the board of AAAASF and helped ensure that office-based surgery in accredited facilities was in fact safe. He noted in a published commentary on a CosmetAssure article that most of the OBSS deaths came from *unaccredited* offices, and his initial study reported on all surgical procedures, not just cosmetic ones. He noted that accreditations vary from among different organizations. Additionally, OBSS may be accredited by AAAASF using its regular or non-Center for Medicare and Medicaid Services (CMS) standards. In contrast, most ASCs and hospitals fall under CMS accreditation standards.^35^

VTE is the most significant untoward sequela and abdominoplasty is the aesthetic procedure most commonly associated with VTE. This was documented in the *Aesthetic Surgery Journal* article “Incidence and Predictors of Venous Thromboembolism in Abdominoplasty,” authored by Keyes et al based on data obtained from IBQAP.^36^ Patient sex, duration of anesthesia and surgery, type of anesthesia, type of additional procedures, and number of procedures did not appear to influence the VTE risk. The article highlighted that, in this outpatient cohort, over 95% of the VTEs seen were in patients whose Caprini risk was between 2 and 8. Per the guidelines, these patients would not be recommended to have chemoprophylaxis. It reinforced to practicing physicians that some procedures may carry a higher risk. Further investigation into proper VTE prophylaxis is warranted.

In a published commentary about this article, Grotting et al found some supportive parallels to that study as well as some differences from the CosmetAssure data.^37,38^ Both showed that patients with a body mass index greater than 25 kg/m^2^ have a greater risk factor for postoperative infection and VTE in aesthetic surgery. The CosmetAssure study found that abdominoplasty combined with liposuction had a higher rate of VTE compared to abdominoplasty alone as compared to the Keyes article. The database also suggested an increased risk of VTE with an increasing number of procedures performed. In summary, both studies showed there to be an increased risk for VTE with abdominoplasty focusing the surgeon on important factors that help guide them in choices to decrease VTE risk for their patients. The studies recognize the limitations of the Caprini Risk Assessment Module in this cohort of patients.

AAAASF regulations, requirements, and database have been used by organized plastic surgery in its presentation to the FDA for processing and evaluating two pre-market approval applications for silicone gel-filled breast prostheses. At the panel, Dr. McGuire stressed the reporting of complications by surgeons semi-annually highlighting the accumulation of accurate data from these accredited facilities. The review of 246,552 breast implant procedures certainly played a role in their approval by the FDA in 2006.

## THE ELDERLY

AAAASF data have also been used to assess surgical risk in the elderly. In 2010, there were over 40 million people over 65, a growth of 8% from 2010. A comparable growth was seen in octogenarians.

Cosmetic surgery in the elderly has been shown to be safe.^39^ The complication rate in this cohort was comparable to previously published data.^40^

In “Commentary on: Safety of Cosmetic Procedures in Elderly and Octogenarian Patients,”^41^ Dr. Singer stated that safety is a priority regardless of patient age. “Elderly patients require greater screening, higher medical clearance, appropriate selectivity of the facility, consideration for hospitalization, and assessment of the social situation for care following the procedures.” He stressed the importance of board-certified or board-eligible plastic surgeons, as well as operating in accredited or licensed surgical facilities.

He reiterated that only 27 states require any accreditation of office-based surgical facilities and called for the public, the media, medical boards, and state legislatures to demand that all surgical procedures (plastic surgery and all other types) other than those under just local anesthesia or minimal oral tranquilization be performed only in licensed or accredited surgical facilities.^41^

## INTERNATIONAL ACCREDITATION

AAAASF received a request to accredit a facility in Australia in 1996. The Board elected to pursue that concept of international accreditation. To further advance accreditation globally, Surgery Facilities Resources, Inc. (SFR), a subsidiary organization to AAAASF, was created in 2005 to offer and build this program. The first President of SFR was Dr. McGuire who was then followed by Dr. Iverson.

In 2006, in efforts to promote increased awareness of patient safety internationally, the International Society of Aesthetic Plastic Surgery (ISAPS) contracted with Surgery Facilities Resources (SFR) to offer a program of accreditation internationally. As a new ISAPS member benefit, surgical facilities could now be internationally accredited through SFR. This international program was presented to the ISAPS membership by Dr. Joao Carlos Sampaio Goes, from Sao Paulo, Brazil. Dr. Goes’ support was critical to the success of the international program.

In 2009, Surgery Facility Resources (SFR) was renamed as AAAASF International (AAAASF-I) to provide resources to surgery centers to enhance patient safety around the world, recognizing that the bar of safety needed to be raised internationally. There were presentations at international medical meetings and to associations regarding patient safety and medical tourism.^40^ The guidelines of AAAASF were modified to be used internationally, adapting to the specific needs of different countries. The standards cover 10 essential areas of patient safety: general environment, operating room environment, recovery room environment, general safety, medications, medical records, quality assessment and improvements, personnel, governance and anesthesia. Accreditation levels are based on the type of anesthesia that is used in a facility.^42,43^

AAAASF-I, like AAAASF, requires 100% compliance with all standards.^44^ An advisory committee with representation from other countries was established. One of the goals of the program was to develop a global workforce of trained SFR inspectors from various countries. AAAASF-I has continued to collaborate with national government agencies including ministries of tourism and ministries of health to improve patient safety.

AAAASF-I in 2015 received a 4-year approval from the International Society for Quality in Health Care (ISQUA) for certification of international facilities in 2016, which was felt to be beneficial to compete effectively in international accreditation.

In October 2017, Drs Nahai and Singer coordinated and presented an AAAASF-I safety panel at the ISAPS International Congress in Kyoto urging ISAPS to follow ASAPS and ASPS in requiring its members to operate only in accredited or license facilities to raise the bar of world-wide patient safety. That effort has been supported by the ISAPS President, Dr. Renato Saltz.

AAAASF-I has made great inroads into international accreditation through the efforts of Drs. Iverson, Colon, McGuire, Keyes, Nahai, Singer, and Alberto Arguello from Costa Rica.

## LEADERSHIP

The first 4 presidents of AAAAPSF/AAAASF were also at some point president of ASAPS and 7 of the 13 were president of ASAPS, ASERF, or both (Table 4). All the AAAASF presidents were members of ASAPS.

**Table 4.** Presidents of AAAAPSF and AAAASF

Prior Presidents
Edward Truppman (AAAAPSF), 1980-1989
Gus Colon (AAAAPSF), 1989-1994
Dan Morello, 1994-1998
Robert Singer, 1998-2000
Ronald Iverson, 2000-2002
Michael McGuire, 2002-2004
Jim Yates, 2004-2006
Alan Gold, 2006-2008
Larry Reed, 2008-2010
Harlan Pollock, 2010-2012
Geoffrey Keyes, 2012-2014
Foad Nahai, 2014-2016
David Watts, 2016-2018

AAAAPSF, The American Association for Accreditation of Ambulatory Plastic Facilities.

Through the years, AAAASF has been blessed to have an excellent hard-working staff; many have been with the organization for many years.

## FUTURE

The migration of care to nonhospital settings has continued and AAAASF has progressively grown and thrived.^45^ It is anticipated that trend will continue.

Improving safety is a never-ending task and a priority for ethical physicians. AAAASF has played a significant role in the area in ambulatory surgery, especially plastic surgery. The future holds safer, better results which will be achieved through continued modifications of standards, research, evidence-based medicine,^46^ better data about outcomes and by identifying the root causes of untoward results. This has been spear-headed by AAAASF and will come from organizations like AAAASF, ASERF, ASAPS, ASPS, and the EF. We are confident that AAAASF which has undergone a structural change to address business development areas beyond ambulatory surgery under Dr. Watts will continue to be in the forefront of safety in plastic surgery.

## Dedication

In honor of all those leaders of AAAASF who have brought it to today’s level of success.

## Disclosures

The authors declared no potential conflicts of interest with respect to the research, authorship, and publication of this article.

## Funding

The authors received no financial support for the research, authorship, and publication of this article.
