# Corrigendum

**DOI:** 10.1093/asjof/ojz013

**Published:** 2019-05-08

Corrigendum to “American Association for Accreditation of Ambulatory Surgical Facilities (AAAASF) History: Its Role in Plastic Surgery Safety” *Aesthetic Surgery Journal Open Forum*, Volume 1, Issue 2, June 2019, ojz008, https://doi.org/10.1093/asjof/ojz008.

In this article, there was a typo in the original title, which read “American Association for Accreditation of Ambulatory Plastic Facilities (AAAASF) History: Its Role in Plastic Surgery Safety”. The title has been corrected to “American Association for Accreditation of Ambulatory Surgical Facilities (AAAASF) History: Its Role in Plastic Surgery Safety”.

