# Corrigendum to: American Association for Accreditation of Ambulatory Surgery Facilities (AAAASF) History: Its Role in Plastic Surgery Safety

**DOI:** 10.1093/asjof/ojz032

**Published:** 2019-11-25

**Authors:** Robert Singer, Geoffrey R Keyes, Foad Nahai

Corrigendum to “American Association for Accreditation of Ambulatory Surgery Facilities (AAAASF) History: Its Role in Plastic Surgery Safety” Aesthetic Surgery Journal Open Forum, Volume 1, Issue 2, June 2019, ojz008, https://doi.org/10.1093/asjof/ojz008. In this article, there was a typo in the title, which read “American Association for Accreditation of Ambulatory Surgical Facilities (AAAASF) History: Its Role in Plastic Surgery Safety”. The title has been corrected to “American Association for Accreditation of Ambulatory Surgery Facilities (AAAASF) History: Its Role in Plastic Surgery Safety”.

